# Bis(tetra­phenyl­phospho­nium) bis­[*N*-(trifluoro­methyl­sulfon­yl)dithio­carbimato(2−)-κ^2^ *S*,*S*′]zincate(II)

**DOI:** 10.1107/S160053681105207X

**Published:** 2011-12-10

**Authors:** Paula S. Pinto, Mayura M. M. Rubinger, Silvana Guilardi, Drielly A. Paixão, Marcelo R. L. Oliveira

**Affiliations:** a Departamento de Química – UFV, Viçosa MG, Brazil; b Instituto de Química – UFU, Uberlândia, MG, Brazil

## Abstract

The title salt, (C_24_H_20_P)_2_[Zn(C_2_F_3_NO_2_S_3_)_2_], consists of a complex dianion and two tetra­phenyl­phospho­nium cations. The Zn^II^ ion displays a distorted tetra­hedral coordination environment with four S atoms from two *S*,*S*′-chelated *N*-(trifluoro­methyl­sulfonyl­)dithio­carbimate anions. In the crystal, besides the ionic inter­action of the oppositely charged ions, inter­molecular C—H⋯O inter­actions between cations and anions are observed. One of the cations inter­acts with an inversion-related equivalent by π–π stacking between phenyl rings, with a centroid–centroid distance of 3.932 (4) Å.

## Related literature

For the anti­fungal and vulcanization activities and crystal structures of dithio­carbimato complexes, see: Amim *et al.* (2011 ▶); Alves *et al.* (2009 ▶); Mariano *et al.* (2007 ▶); Oliveira *et al.* (2007 ▶); Perpétuo *et al.* (2003 ▶). For further synthetic details, see: Franca *et al.* (2006 ▶). For other literature related to fungicides, see: Hogarth (2005 ▶).
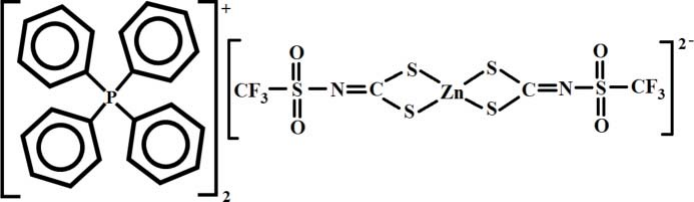


## Experimental

### 

#### Crystal data

(C_24_H_20_P)_2_[Zn(C_2_F_3_NO_2_S_3_)_2_]
*M* *_r_* = 1190.53
Monoclinic, 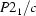

*a* = 8.8461 (1) Å
*b* = 29.1869 (5) Å
*c* = 20.6963 (3) Å
β = 93.578 (1)°
*V* = 5333.17 (13) Å^3^
*Z* = 4
Mo *K*α radiation
μ = 0.82 mm^−1^
*T* = 295 K
0.42 × 0.18 × 0.12 mm

#### Data collection

Nonius KappaCCD diffractometer
Absorption correction: multi-scan (*SORTAV*; Blessing, 1995 ▶) *T*_min_ = 0.758, *T*_max_ = 0.950
57739 measured reflections
11977 independent reflections
8307 reflections with *I* > 2σ(*I*)
*R*_int_ = 0.082

#### Refinement

*R*[*F* ^2^ > 2σ(*F* ^2^)] = 0.058
*wR*(*F* ^2^) = 0.169
*S* = 1.05
11977 reflections
646 parameters
H-atom parameters constrained
Δρ_max_ = 1.18 e Å^−3^
Δρ_min_ = −1.66 e Å^−3^

### 

Data collection: *COLLECT* (Nonius, 2000 ▶); cell refinement: *DENZO-SMN* (Otwinowski & Minor, 1997 ▶); data reduction: *DENZO-SMN*; program(s) used to solve structure: *SHELXS97* (Sheldrick, 2008 ▶); program(s) used to refine structure: *SHELXL97* (Sheldrick, 2008 ▶); molecular graphics: *ORTEP-3 for Windows* (Farrugia, 1997 ▶); software used to prepare material for publication: *WinGX* (Farrugia, 1999 ▶).

## Supplementary Material

Crystal structure: contains datablock(s) I, global. DOI: 10.1107/S160053681105207X/pk2371sup1.cif

Structure factors: contains datablock(s) I. DOI: 10.1107/S160053681105207X/pk2371Isup2.hkl

Additional supplementary materials:  crystallographic information; 3D view; checkCIF report

## Figures and Tables

**Table:** 

Zn—S1	2.3346 (10)
Zn—S2	2.3340 (10)
Zn—S4	2.3376 (9)
Zn—S5	2.3566 (10)

**Table:** 

S2—Zn—S1	77.84 (3)
S4—Zn—S5	77.63 (3)

**Table 2:** Hydrogen-bond geometry (Å, °)

*D*—H⋯*A*	*D*—H	H⋯*A*	*D*⋯*A*	*D*—H⋯*A*
C9—H9⋯O1	0.93	2.68	3.424 (4)	138
C31—H31⋯O4	0.93	2.62	3.470 (4)	153
C33—H33⋯O2^i^	0.93	2.47	3.283 (4)	145

Symmetry code: (i) 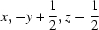.

**Fig. 1.**
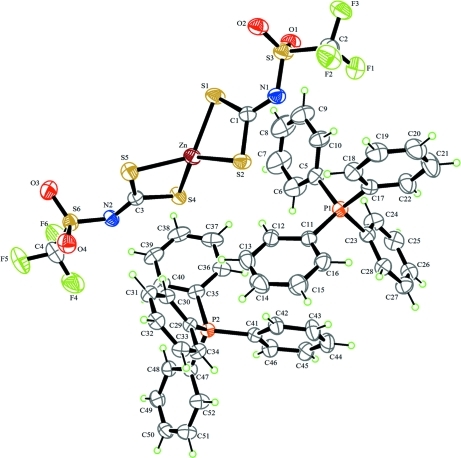
Representation of the title compound, with displacement ellipsoids drawn at the 30% probability level.

## supplementary crystallographic information

### Comment

We became interested in the syntheses and characterization of dithiocarbimato-metal complexes due to their similarities with dithiocarbamato complexes, which are important fungicides (Hogarth, 2005). While the dithiocarbamato- metal(II) are neutral substances, the analogous dithiocarbimato-complexes are necessarily anionic species. Thus, the choice of metallic ion, active counter ions or the use of different *R* groups on the dithiocarbimate structures could improve and/or modulate the antifungal activity.

The title complex is a new member of the class of Zn complexes with general formula [Zn(RSO_2_N═CS_2_)_2_]^2-^ (Amim *et al.*, 2011; Alves *et al.*, 2009; Mariano *et al.*, 2007; Perpétuo *et al.*, 2003). The literature describes only one complex of this class having an alkyl group (methyl) attached to the SO_2_ moiety (Oliveira *et al.*, 2007). The asymmetric unit of the title compound is shown in Fig. 1, and consists of one [Zn(CF_3_SO_2_N═CS_2_)_2_]^2-^ anion and two Ph_4_P^+^ cations. The Zn^II^ ion is coordinated by two *S*,*S*'-chelated *N*-trifluoromethylsulfonyldithiocarbimate ligands, resulting in a slightly distorted ZnS_4_ tetrahedral geometry. Due to the formation of the two ZnS_2_C four membered rings, the two S—Zn—S angles containing both sulfur atoms of the same ligand are significantly smaller than those containing the sulfur atoms from two ligands (Table 1). The dihedral angle between the two ZnS_2_C four membered rings [87.40 (4)°] is greater than that found in the salt (Ph_4_P)_2_[Zn(CH_3_SO_2_N═CS_2_)_2_] of 79.1 (1)° (Oliveira *et al.*, 2007). The C—S [average value of 1.731 Å] and C═N bond distances [1.315 (4) and 1.314 (4) Å] have double bond character. This behavior indicates that the electron density is delocalized over the entire NCS_2_ moiety. The S1–C1–N1 and S5–C3–N2 angles are significantly greater than S2–C1–N1 and S4–C3–N2 due to the repulsive interactions between the two SO_2_CF_3_ groups and the S1 and S5 atoms, respectively, which are *cis* in relation to the C1–N1 and C3–N2 bonds. Similar behavior is observed for other zinc complexes with dithiocarbimato ligands (Amim *et al.*, 2011; Alves *et al.*, 2009; Oliveira *et al.*, 2007; Perpétuo *et al.*, 2003). The torsion angles of C1–N1–S3–C2 and C3–N2–S6–C4 describing the conformation of the dithiocarbimato ligands are -175.9 (3)° and 180.0 (3)°, respectively. These angles are -67.1 (2)° and 159.6 (2)° in the compound (Ph_4_P)_2_[Zn(CH_3_SO_2_N═CS_2_)_2_] (Oliveira *et al.*, 2007), probably due to the larger size of the CF_3_ group and the intermolecular interaction present in the title compound.

In both tetraphenylphosphonium cations, the P–C bond lengths range from 1.785 (3) to 1.803 (3) Å, the C–P–C angles range from 106.6 (2) to 112.1 (1)°, and the P atoms display slightly distorted tetrahedral geometry. The arrangement of molecules is mainly determined by the electrostatic interactions between oppositely charged units. Moreover, there are intermolecular C—H···O interactions between cations and anions (Table 2) and π-π stacking between phenyl rings on inversion related (1-x, -y, 2-z) cations.

### Experimental

The potassium trifluoromethylsulfonyldithiocarbimate dihydrate was prepared in dimethylformamide from trifluoromethanesulfonamide as described in the literature (Franca *et al.*, 2006). The title compound was prepared in 1:1 (10 ml) methanol:water by the reaction of zinc acetate dihydrate (1.0 mmol) with trifluoromethylsulfonyldithiocarbimate dihydrate (2.0 mmol) and tetraphenylphosphanium chloride (2.0 mmol). The reaction mixture was stirred for 1 h at room temperature. The white solid obtained was filtered, washed with distilled water and dried under reduced pressure. The title compound is insoluble in water but soluble in chloroform, methanol and dichloromethane. Suitable crystals were obtained after slow evaporation of the solution in dichloromethene:ethanol 2:1 mixture. *M*.p. 161.5–163.3 °C. IR (selected bands, cm^-1^): 1395, 1378 *v*(C═N); 1315, 1183 (*v*CF_3_), 1298 *v*_as_(SO_2_); 1110 *v_sym_*(SO_2_); 951 *v*_as_(CS_2_) and 323 *v*(ZnS). ^13^C NMR (dithiocarbimate anion signals) (δ): 119.3 (q, CF_3_, *J* = 317,2 Hz), 226.3 (s, C═N). All spectra (IR, ^1^H NMR and ^13^C NMR) showed the expected signals for the tetraphenylphosphonium cation.

### Refinement

H atoms were geometrically positioned (C–H 0.93 Å) and refined as riding, with *U*_iso_(H) = 1.2 *U*_eq_ of the parent atom. Anisotropic displacement parameters were made equal for the S3, O2 and O1 atoms, using the SHELXL-97 EADP constraint.  Reflections (-1 1 2) and (0 4 2) were omitted because they were partially obscured by the beamstop.

### Crystal data

**Table:**

(C_24_H_20_P)_2_[Zn(C_2_F_3_NO_2_S_3_)_2_]	*F*(000) = 2432
*M**_r_* = 1190.53	*D*_x_ = 1.483 Mg m^−^^3^
Monoclinic, *P*2_1_/*c*	Mo *K*α radiation, λ = 0.71073 Å
*a* = 8.8461 (1) Å	Cell parameters from 80040 reflections
*b* = 29.1869 (5) Å	θ = 2.9–27.5°
*c* = 20.6963 (3) Å	µ = 0.82 mm^−^^1^
β = 93.578 (1)°	*T* = 295 K
*V* = 5333.17 (13) Å^3^	Prism, colourless
*Z* = 4	0.42 × 0.18 × 0.12 mm

### Data collection

**Table:**

Nonius KappaCCD diffractometer	11977 independent reflections
Radiation source: Enraf Nonius FR590	8307 reflections with *I* > 2σ(*I*)
graphite	*R*_int_ = 0.082
Detector resolution: 9 pixels mm^-1^	θ_max_ = 27.5°, θ_min_ = 2.9°
CCD rotation images, thick slices scans	*h* = −11→7
Absorption correction: multi-scan (*SORTAV*; Blessing, 1995)	*k* = −37→37
*T*_min_ = 0.758, *T*_max_ = 0.950	*l* = −26→26
57739 measured reflections	

### Refinement

**Table:**

Refinement on *F*^2^	Primary atom site location: structure-invariant direct methods
Least-squares matrix: full	Secondary atom site location: difference Fourier map
*R*[*F*^2^ > 2σ(*F*^2^)] = 0.058	Hydrogen site location: inferred from neighbouring sites
*wR*(*F*^2^) = 0.169	H-atom parameters constrained
*S* = 1.05	*w* = 1/[σ^2^(*F*_o_^2^) + (0.0872*P*)^2^ + 2.420*P*] where *P* = (*F*_o_^2^ + 2*F*_c_^2^)/3
11977 reflections	(Δ/σ)_max_ = 0.003
646 parameters	Δρ_max_ = 1.18 e Å^−^^3^
0 restraints	Δρ_min_ = −1.66 e Å^−^^3^

### Special details

**Table:**

Geometry. All e.s.d.'s (except the e.s.d. in the dihedral angle between two l.s. planes) are estimated using the full covariance matrix. The cell e.s.d.'s are taken into account individually in the estimation of e.s.d.'s in distances, angles and torsion angles; correlations between e.s.d.'s in cell parameters are only used when they are defined by crystal symmetry. An approximate (isotropic) treatment of cell e.s.d.'s is used for estimating e.s.d.'s involving l.s. planes.
Refinement. Refinement of *F*^2^ against ALL reflections. The weighted *R*-factor *wR* and goodness of fit *S* are based on *F*^2^, conventional *R*-factors *R* are based on *F*, with *F* set to zero for negative *F*^2^. The threshold expression of *F*^2^ > σ(*F*^2^) is used only for calculating *R*-factors(gt) *etc*. and is not relevant to the choice of reflections for refinement. *R*-factors based on *F*^2^ are statistically about twice as large as those based on *F*, and *R*- factors based on ALL data will be even larger.

### Fractional atomic coordinates and isotropic or equivalent isotropic displacement parameters (Å^2^)

**Table:**

	*x*	*y*	*z*	*U*_iso_*/*U*_eq_	
Zn	0.76875 (4)	0.258126 (13)	1.300410 (19)	0.05905 (14)	
S1	0.76765 (13)	0.32502 (3)	1.36222 (5)	0.0744 (3)	
S2	0.89252 (11)	0.30995 (3)	1.23596 (4)	0.0636 (2)	
S3	0.88481 (12)	0.42771 (3)	1.34592 (5)	0.0706 (2)	
S4	0.57897 (9)	0.20559 (3)	1.26862 (5)	0.0615 (2)	
S5	0.87243 (10)	0.18826 (3)	1.33924 (5)	0.0658 (2)	
S6	0.77346 (10)	0.08409 (3)	1.33543 (4)	0.0600 (2)	
P1	0.58019 (9)	0.36494 (3)	1.07700 (4)	0.0529 (2)	
P2	0.41864 (8)	0.12861 (3)	1.04277 (4)	0.04373 (18)	
F1	0.8570 (4)	0.48605 (10)	1.25094 (14)	0.1171 (10)	
F2	1.0842 (3)	0.47099 (9)	1.28085 (14)	0.1085 (9)	
F3	0.9532 (4)	0.51320 (8)	1.33994 (13)	0.1030 (9)	
F4	0.6206 (4)	0.03089 (10)	1.25260 (17)	0.1194 (10)	
F5	0.7129 (4)	−0.00242 (9)	1.33781 (18)	0.1227 (11)	
F6	0.5171 (3)	0.03994 (10)	1.34194 (19)	0.1285 (11)	
N1	0.9004 (3)	0.39051 (10)	1.29069 (13)	0.0605 (7)	
N2	0.6657 (3)	0.12278 (9)	1.30365 (13)	0.0559 (6)	
O1	0.7346 (3)	0.43889 (9)	1.36095 (12)	0.0706 (2)	
O2	0.9950 (3)	0.42280 (8)	1.39932 (12)	0.0706 (2)	
O3	0.7885 (4)	0.08521 (10)	1.40437 (13)	0.0839 (8)	
O4	0.9063 (3)	0.07468 (9)	1.30165 (13)	0.0758 (7)	
C1	0.8577 (3)	0.34775 (11)	1.29750 (15)	0.0537 (7)	
C2	0.9492 (5)	0.47687 (14)	1.3012 (2)	0.0765 (11)	
C3	0.7047 (3)	0.16624 (11)	1.30466 (14)	0.0503 (7)	
C4	0.6486 (5)	0.03583 (14)	1.3155 (3)	0.0853 (12)	
C5	0.4940 (4)	0.36160 (13)	1.15287 (17)	0.0638 (9)	
C6	0.3858 (6)	0.32851 (19)	1.1625 (2)	0.0986 (15)	
H6	0.3616	0.3069	1.1305	0.118*	
C7	0.3137 (7)	0.3277 (3)	1.2198 (3)	0.128 (2)	
H7	0.2419	0.3052	1.2268	0.153*	
C8	0.3489 (7)	0.3605 (3)	1.2668 (3)	0.125 (2)	
H8	0.2972	0.3608	1.3046	0.151*	
C9	0.4583 (6)	0.3920 (2)	1.2581 (2)	0.1071 (17)	
H9	0.4846	0.413	1.2908	0.129*	
C10	0.5303 (4)	0.39325 (15)	1.20132 (18)	0.0744 (10)	
H10	0.6037	0.4154	1.1953	0.089*	
C11	0.6968 (4)	0.31543 (11)	1.06493 (16)	0.0558 (8)	
C12	0.6925 (4)	0.27720 (12)	1.10471 (17)	0.0664 (9)	
H12	0.6242	0.2759	1.1371	0.08*	
C13	0.7902 (6)	0.24120 (13)	1.0960 (2)	0.0816 (13)	
H13	0.7874	0.2157	1.1228	0.098*	
C14	0.8906 (6)	0.24230 (15)	1.0487 (3)	0.0889 (14)	
H14	0.9562	0.2178	1.0436	0.107*	
C15	0.8946 (5)	0.27990 (17)	1.0084 (2)	0.0878 (12)	
H15	0.9617	0.2805	0.9755	0.105*	
C16	0.7991 (4)	0.31657 (14)	1.01673 (18)	0.0693 (9)	
H16	0.8034	0.3421	0.99	0.083*	
C17	0.7020 (4)	0.41366 (11)	1.07582 (15)	0.0537 (7)	
C18	0.8430 (4)	0.41156 (14)	1.11035 (18)	0.0672 (9)	
H18	0.8731	0.3851	1.1326	0.081*	
C19	0.9370 (5)	0.44945 (15)	1.1109 (2)	0.0793 (11)	
H19	1.0305	0.4485	1.1341	0.095*	
C20	0.8941 (6)	0.48820 (16)	1.0781 (2)	0.0939 (14)	
H20	0.9586	0.5134	1.0787	0.113*	
C21	0.7568 (6)	0.49019 (16)	1.0441 (3)	0.1064 (17)	
H21	0.7281	0.5167	1.0216	0.128*	
C22	0.6601 (5)	0.45280 (13)	1.0432 (2)	0.0794 (11)	
H22	0.5664	0.4543	1.0203	0.095*	
C23	0.4320 (3)	0.37039 (11)	1.01393 (16)	0.0536 (7)	
C24	0.2849 (4)	0.38278 (15)	1.0277 (2)	0.0750 (10)	
H24	0.2615	0.3879	1.0703	0.09*	
C25	0.1748 (4)	0.38742 (15)	0.9783 (2)	0.0790 (11)	
H25	0.0768	0.3953	0.9879	0.095*	
C26	0.2071 (4)	0.38068 (13)	0.9152 (2)	0.0698 (10)	
H26	0.1316	0.3839	0.8822	0.084*	
C27	0.3520 (4)	0.36912 (14)	0.90089 (19)	0.0698 (9)	
H27	0.3748	0.3649	0.858	0.084*	
C28	0.4642 (4)	0.36372 (12)	0.95011 (16)	0.0616 (8)	
H28	0.5617	0.3556	0.9401	0.074*	
C29	0.6183 (3)	0.12592 (9)	1.06577 (13)	0.0418 (6)	
C30	0.6682 (3)	0.12508 (10)	1.13043 (14)	0.0475 (6)	
H30	0.5996	0.129	1.1623	0.057*	
C31	0.8211 (3)	0.11840 (11)	1.14776 (15)	0.0525 (7)	
H31	0.8553	0.1173	1.1911	0.063*	
C32	0.9212 (3)	0.11350 (11)	1.09979 (16)	0.0527 (7)	
H32	1.0234	0.1087	1.1112	0.063*	
C33	0.8732 (3)	0.11551 (11)	1.03584 (16)	0.0528 (7)	
H33	0.9427	0.1127	1.0042	0.063*	
C34	0.7213 (3)	0.12172 (11)	1.01815 (15)	0.0507 (7)	
H34	0.6883	0.1231	0.9746	0.061*	
C35	0.3211 (3)	0.13903 (11)	1.11472 (15)	0.0510 (7)	
C36	0.2516 (4)	0.18093 (13)	1.12335 (18)	0.0625 (8)	
H36	0.2526	0.2033	1.0914	0.075*	
C37	0.1799 (4)	0.18924 (16)	1.1806 (2)	0.0781 (12)	
H37	0.1345	0.2175	1.1871	0.094*	
C38	0.1766 (4)	0.15591 (19)	1.22685 (19)	0.0811 (13)	
H38	0.126	0.1614	1.2642	0.097*	
C39	0.2459 (4)	0.11501 (17)	1.21909 (17)	0.0741 (11)	
H39	0.2439	0.0929	1.2514	0.089*	
C40	0.3192 (3)	0.10597 (13)	1.16364 (15)	0.0588 (8)	
H40	0.3673	0.078	1.1588	0.071*	
C41	0.3778 (3)	0.17355 (10)	0.98562 (14)	0.0489 (7)	
C42	0.4799 (4)	0.20877 (11)	0.97580 (18)	0.0634 (9)	
H42	0.5729	0.2095	0.9994	0.076*	
C43	0.4427 (5)	0.24267 (13)	0.9309 (2)	0.0813 (12)	
H43	0.5116	0.2659	0.9238	0.098*	
C44	0.3046 (5)	0.24214 (14)	0.8969 (2)	0.0821 (12)	
H44	0.2797	0.2652	0.8672	0.099*	
C45	0.2028 (5)	0.20768 (15)	0.90656 (19)	0.0764 (11)	
H45	0.1087	0.2079	0.8838	0.092*	
C46	0.2388 (4)	0.17285 (13)	0.94965 (17)	0.0637 (9)	
H46	0.171	0.149	0.9548	0.076*	
C47	0.3599 (3)	0.07588 (10)	1.00384 (14)	0.0472 (6)	
C48	0.2587 (4)	0.04559 (11)	1.02871 (16)	0.0559 (7)	
H48	0.2166	0.0519	1.0678	0.067*	
C49	0.2205 (4)	0.00612 (12)	0.99541 (19)	0.0668 (9)	
H49	0.1515	−0.0141	1.012	0.08*	
C50	0.2826 (4)	−0.00369 (12)	0.93823 (19)	0.0652 (9)	
H50	0.257	−0.0307	0.9164	0.078*	
C51	0.3832 (4)	0.02640 (14)	0.91288 (19)	0.0718 (10)	
H51	0.4256	0.0197	0.874	0.086*	
C52	0.4205 (4)	0.06620 (13)	0.94506 (17)	0.0670 (9)	
H52	0.4868	0.0868	0.9275	0.08*	

### Atomic displacement parameters (Å^2^)

**Table:**

	*U*^11^	*U*^22^	*U*^33^	*U*^12^	*U*^13^	*U*^23^
Zn	0.0568 (2)	0.0545 (2)	0.0660 (3)	−0.00555 (17)	0.00531 (18)	−0.00531 (17)
S1	0.0976 (7)	0.0628 (5)	0.0662 (6)	−0.0187 (5)	0.0324 (5)	−0.0122 (4)
S2	0.0715 (6)	0.0615 (5)	0.0595 (5)	−0.0087 (4)	0.0170 (4)	−0.0105 (4)
S3	0.0793 (5)	0.0670 (5)	0.0670 (5)	−0.0189 (4)	0.0169 (4)	−0.0131 (4)
S4	0.0452 (4)	0.0540 (5)	0.0840 (6)	0.0023 (3)	−0.0067 (4)	−0.0026 (4)
S5	0.0505 (5)	0.0657 (5)	0.0792 (6)	−0.0007 (4)	−0.0113 (4)	0.0004 (4)
S6	0.0575 (5)	0.0567 (5)	0.0661 (5)	0.0069 (4)	0.0063 (4)	0.0033 (4)
P1	0.0490 (4)	0.0558 (5)	0.0538 (5)	−0.0078 (4)	0.0028 (3)	−0.0004 (4)
P2	0.0310 (3)	0.0516 (4)	0.0485 (4)	−0.0019 (3)	0.0015 (3)	−0.0016 (3)
F1	0.167 (3)	0.0894 (18)	0.0918 (18)	0.0078 (18)	−0.0169 (19)	0.0137 (14)
F2	0.114 (2)	0.0865 (17)	0.131 (2)	−0.0304 (16)	0.0558 (18)	−0.0042 (16)
F3	0.149 (3)	0.0613 (13)	0.0995 (18)	−0.0193 (15)	0.0172 (17)	−0.0187 (12)
F4	0.129 (2)	0.0855 (18)	0.139 (3)	−0.0078 (17)	−0.028 (2)	−0.0277 (17)
F5	0.119 (2)	0.0566 (14)	0.191 (3)	0.0091 (14)	0.003 (2)	0.0182 (17)
F6	0.0822 (18)	0.0841 (18)	0.223 (4)	−0.0127 (15)	0.042 (2)	0.006 (2)
N1	0.0680 (17)	0.0590 (16)	0.0557 (15)	−0.0102 (13)	0.0132 (13)	−0.0067 (12)
N2	0.0491 (14)	0.0513 (15)	0.0670 (17)	0.0027 (11)	0.0029 (12)	−0.0022 (12)
O1	0.0793 (5)	0.0670 (5)	0.0670 (5)	−0.0189 (4)	0.0169 (4)	−0.0131 (4)
O2	0.0793 (5)	0.0670 (5)	0.0670 (5)	−0.0189 (4)	0.0169 (4)	−0.0131 (4)
O3	0.102 (2)	0.0844 (18)	0.0650 (16)	0.0128 (16)	0.0061 (14)	0.0117 (13)
O4	0.0603 (14)	0.0793 (17)	0.0889 (18)	0.0176 (13)	0.0128 (13)	0.0008 (14)
C1	0.0469 (16)	0.0598 (19)	0.0545 (18)	−0.0038 (14)	0.0030 (14)	−0.0058 (14)
C2	0.097 (3)	0.062 (2)	0.072 (2)	−0.013 (2)	0.008 (2)	−0.0079 (19)
C3	0.0435 (15)	0.0583 (18)	0.0501 (16)	0.0029 (13)	0.0112 (13)	−0.0027 (13)
C4	0.076 (3)	0.060 (2)	0.121 (4)	0.006 (2)	0.009 (3)	0.003 (2)
C5	0.0565 (19)	0.073 (2)	0.062 (2)	−0.0092 (17)	0.0054 (16)	0.0066 (17)
C6	0.090 (3)	0.122 (4)	0.087 (3)	−0.046 (3)	0.023 (2)	−0.005 (3)
C7	0.105 (4)	0.172 (6)	0.110 (4)	−0.057 (4)	0.043 (3)	0.012 (4)
C8	0.110 (4)	0.183 (6)	0.088 (4)	−0.026 (4)	0.045 (3)	0.007 (4)
C9	0.111 (4)	0.142 (5)	0.071 (3)	−0.010 (4)	0.028 (3)	−0.014 (3)
C10	0.067 (2)	0.096 (3)	0.061 (2)	−0.006 (2)	0.0141 (18)	−0.010 (2)
C11	0.0572 (18)	0.0539 (17)	0.0546 (18)	−0.0058 (14)	−0.0099 (15)	−0.0023 (14)
C12	0.077 (2)	0.057 (2)	0.062 (2)	−0.0165 (18)	−0.0148 (18)	0.0035 (16)
C13	0.096 (3)	0.052 (2)	0.092 (3)	−0.008 (2)	−0.035 (3)	0.0041 (19)
C14	0.089 (3)	0.067 (3)	0.106 (3)	0.018 (2)	−0.030 (3)	−0.014 (2)
C15	0.083 (3)	0.093 (3)	0.086 (3)	0.030 (2)	0.000 (2)	0.002 (2)
C16	0.072 (2)	0.074 (2)	0.062 (2)	0.0119 (19)	0.0010 (18)	0.0075 (17)
C17	0.0503 (17)	0.0574 (18)	0.0541 (17)	−0.0048 (14)	0.0080 (14)	−0.0067 (14)
C18	0.0544 (19)	0.076 (2)	0.071 (2)	−0.0137 (17)	0.0012 (16)	0.0005 (18)
C19	0.063 (2)	0.090 (3)	0.085 (3)	−0.026 (2)	0.004 (2)	−0.012 (2)
C20	0.101 (3)	0.076 (3)	0.105 (3)	−0.038 (3)	0.012 (3)	−0.008 (3)
C21	0.109 (4)	0.063 (2)	0.143 (5)	−0.025 (3)	−0.024 (3)	0.016 (3)
C22	0.079 (3)	0.061 (2)	0.096 (3)	−0.0155 (19)	−0.011 (2)	0.005 (2)
C23	0.0469 (16)	0.0531 (17)	0.0607 (19)	−0.0063 (13)	0.0032 (14)	−0.0014 (14)
C24	0.0534 (19)	0.095 (3)	0.077 (2)	0.0010 (19)	0.0082 (18)	−0.008 (2)
C25	0.0465 (19)	0.094 (3)	0.096 (3)	−0.0001 (19)	0.0016 (19)	−0.004 (2)
C26	0.056 (2)	0.065 (2)	0.085 (3)	−0.0047 (16)	−0.0183 (18)	−0.0004 (19)
C27	0.063 (2)	0.079 (2)	0.065 (2)	0.0018 (18)	−0.0080 (17)	−0.0002 (18)
C28	0.0518 (18)	0.071 (2)	0.062 (2)	0.0013 (16)	0.0013 (15)	0.0037 (16)
C29	0.0331 (12)	0.0454 (14)	0.0469 (15)	−0.0032 (11)	0.0020 (11)	−0.0006 (12)
C30	0.0348 (13)	0.0587 (17)	0.0494 (16)	−0.0034 (12)	0.0041 (12)	−0.0051 (13)
C31	0.0399 (14)	0.0650 (19)	0.0520 (17)	−0.0042 (13)	−0.0019 (13)	0.0009 (14)
C32	0.0306 (13)	0.0585 (18)	0.069 (2)	−0.0038 (12)	0.0005 (13)	−0.0011 (15)
C33	0.0365 (14)	0.0634 (19)	0.0599 (19)	−0.0062 (13)	0.0134 (13)	−0.0071 (14)
C34	0.0394 (14)	0.0643 (18)	0.0487 (16)	−0.0062 (13)	0.0043 (12)	−0.0005 (14)
C35	0.0295 (12)	0.071 (2)	0.0521 (17)	−0.0027 (13)	0.0025 (12)	−0.0085 (15)
C36	0.0449 (16)	0.072 (2)	0.071 (2)	−0.0025 (15)	0.0037 (15)	−0.0159 (17)
C37	0.0470 (19)	0.104 (3)	0.083 (3)	0.0028 (19)	0.0070 (18)	−0.037 (2)
C38	0.0469 (19)	0.140 (4)	0.058 (2)	−0.009 (2)	0.0088 (16)	−0.025 (3)
C39	0.0489 (18)	0.122 (3)	0.0514 (19)	−0.011 (2)	0.0017 (15)	−0.002 (2)
C40	0.0377 (15)	0.085 (2)	0.0541 (18)	−0.0022 (15)	0.0026 (13)	0.0011 (16)
C41	0.0399 (14)	0.0518 (16)	0.0545 (17)	0.0022 (12)	−0.0009 (12)	−0.0022 (13)
C42	0.0588 (19)	0.0536 (18)	0.076 (2)	−0.0053 (15)	−0.0123 (17)	0.0027 (16)
C43	0.086 (3)	0.056 (2)	0.100 (3)	−0.0090 (19)	−0.011 (2)	0.019 (2)
C44	0.091 (3)	0.070 (2)	0.083 (3)	0.012 (2)	−0.014 (2)	0.014 (2)
C45	0.064 (2)	0.086 (3)	0.077 (3)	0.012 (2)	−0.0208 (19)	0.006 (2)
C46	0.0435 (16)	0.071 (2)	0.075 (2)	−0.0003 (15)	−0.0071 (16)	0.0052 (18)
C47	0.0342 (13)	0.0515 (16)	0.0554 (17)	−0.0028 (12)	−0.0011 (12)	−0.0014 (13)
C48	0.0513 (17)	0.0577 (18)	0.0594 (18)	−0.0017 (14)	0.0090 (14)	0.0051 (15)
C49	0.067 (2)	0.0517 (18)	0.082 (2)	−0.0154 (16)	0.0069 (19)	0.0053 (17)
C50	0.068 (2)	0.0477 (17)	0.079 (2)	−0.0017 (15)	−0.0035 (19)	−0.0047 (16)
C51	0.067 (2)	0.076 (2)	0.073 (2)	−0.0100 (19)	0.0159 (18)	−0.0179 (19)
C52	0.0578 (19)	0.076 (2)	0.068 (2)	−0.0235 (17)	0.0161 (16)	−0.0166 (18)

### Geometric parameters (Å, °)

**Table:**

Zn—S1	2.3346 (10)	C20—C21	1.366 (7)
Zn—S2	2.3340 (10)	C20—H20	0.93
Zn—S4	2.3376 (9)	C21—C22	1.386 (6)
Zn—S5	2.3566 (10)	C21—H21	0.93
S1—C1	1.733 (3)	C22—H22	0.93
S2—C1	1.727 (3)	C23—C28	1.382 (5)
S3—O1	1.421 (3)	C23—C24	1.397 (5)
S3—O2	1.435 (3)	C24—C25	1.375 (5)
S3—N1	1.589 (3)	C24—H24	0.93
S3—C2	1.818 (4)	C25—C26	1.369 (6)
S4—C3	1.735 (3)	C25—H25	0.93
S5—C3	1.730 (3)	C26—C27	1.375 (5)
S6—O3	1.425 (3)	C26—H26	0.93
S6—O4	1.431 (3)	C27—C28	1.386 (5)
S6—N2	1.593 (3)	C27—H27	0.93
S6—C4	1.821 (5)	C28—H28	0.93
P1—C17	1.785 (3)	C29—C30	1.383 (4)
P1—C5	1.790 (4)	C29—C34	1.389 (4)
P1—C23	1.798 (3)	C30—C31	1.391 (4)
P1—C11	1.802 (3)	C30—H30	0.93
P2—C41	1.788 (3)	C31—C32	1.379 (4)
P2—C35	1.793 (3)	C31—H31	0.93
P2—C47	1.799 (3)	C32—C33	1.366 (4)
P2—C29	1.802 (3)	C32—H32	0.93
F1—C2	1.309 (5)	C33—C34	1.383 (4)
F2—C2	1.303 (5)	C33—H33	0.93
F3—C2	1.328 (4)	C34—H34	0.93
F4—C4	1.319 (6)	C35—C36	1.385 (5)
F5—C4	1.323 (5)	C35—C40	1.400 (5)
F6—C4	1.321 (5)	C36—C37	1.399 (5)
N1—C1	1.314 (4)	C36—H36	0.93
N2—C3	1.314 (4)	C37—C38	1.367 (7)
C5—C6	1.383 (5)	C37—H37	0.93
C5—C10	1.386 (5)	C38—C39	1.356 (6)
C6—C7	1.381 (7)	C38—H38	0.93
C6—H6	0.93	C39—C40	1.379 (5)
C7—C8	1.387 (8)	C39—H39	0.93
C7—H7	0.93	C40—H40	0.93
C8—C9	1.356 (8)	C41—C42	1.391 (4)
C8—H8	0.93	C41—C46	1.397 (4)
C9—C10	1.372 (6)	C42—C43	1.382 (5)
C9—H9	0.93	C42—H42	0.93
C10—H10	0.93	C43—C44	1.372 (6)
C11—C12	1.389 (5)	C43—H43	0.93
C11—C16	1.389 (5)	C44—C45	1.373 (6)
C12—C13	1.380 (6)	C44—H44	0.93
C12—H12	0.93	C45—C46	1.377 (5)
C13—C14	1.362 (7)	C45—H45	0.93
C13—H13	0.93	C46—H46	0.93
C14—C15	1.381 (7)	C47—C48	1.381 (4)
C14—H14	0.93	C47—C52	1.389 (4)
C15—C16	1.381 (6)	C48—C49	1.374 (5)
C15—H15	0.93	C48—H48	0.93
C16—H16	0.93	C49—C50	1.366 (5)
C17—C22	1.366 (5)	C49—H49	0.93
C17—C18	1.400 (5)	C50—C51	1.377 (5)
C18—C19	1.383 (5)	C50—H50	0.93
C18—H18	0.93	C51—C52	1.369 (5)
C19—C20	1.362 (7)	C51—H51	0.93
C19—H19	0.93	C52—H52	0.93
			
S2—Zn—S1	77.84 (3)	C19—C20—C21	120.2 (4)
S2—Zn—S4	128.21 (4)	C19—C20—H20	119.9
S1—Zn—S4	132.48 (4)	C21—C20—H20	119.9
S2—Zn—S5	124.69 (4)	C20—C21—C22	120.1 (4)
S1—Zn—S5	123.49 (4)	C20—C21—H21	119.9
S4—Zn—S5	77.63 (3)	C22—C21—H21	119.9
C1—S1—Zn	83.04 (11)	C17—C22—C21	120.2 (4)
C1—S2—Zn	83.17 (11)	C17—C22—H22	119.9
O1—S3—O2	117.00 (15)	C21—C22—H22	119.9
O1—S3—N1	116.02 (16)	C28—C23—C24	118.8 (3)
O2—S3—N1	113.61 (16)	C28—C23—P1	119.8 (2)
O1—S3—C2	104.9 (2)	C24—C23—P1	121.4 (3)
O2—S3—C2	104.58 (19)	C25—C24—C23	120.0 (4)
N1—S3—C2	97.43 (17)	C25—C24—H24	120
C3—S4—Zn	83.31 (11)	C23—C24—H24	120
C3—S5—Zn	82.82 (11)	C26—C25—C24	121.0 (4)
O3—S6—O4	117.73 (18)	C26—C25—H25	119.5
O3—S6—N2	114.39 (16)	C24—C25—H25	119.5
O4—S6—N2	115.02 (15)	C25—C26—C27	119.6 (3)
O3—S6—C4	105.2 (2)	C25—C26—H26	120.2
O4—S6—C4	104.2 (2)	C27—C26—H26	120.2
N2—S6—C4	96.67 (18)	C26—C27—C28	120.3 (4)
C17—P1—C5	110.23 (16)	C26—C27—H27	119.9
C17—P1—C23	109.44 (15)	C28—C27—H27	119.9
C5—P1—C23	108.09 (16)	C23—C28—C27	120.3 (3)
C17—P1—C11	106.60 (15)	C23—C28—H28	119.8
C5—P1—C11	110.89 (17)	C27—C28—H28	119.8
C23—P1—C11	111.59 (15)	C30—C29—C34	120.1 (3)
C41—P2—C35	109.70 (15)	C30—C29—P2	120.3 (2)
C41—P2—C47	106.90 (14)	C34—C29—P2	119.5 (2)
C35—P2—C47	112.13 (14)	C29—C30—C31	119.9 (3)
C41—P2—C29	111.16 (13)	C29—C30—H30	120.1
C35—P2—C29	107.61 (13)	C31—C30—H30	120.1
C47—P2—C29	109.39 (13)	C32—C31—C30	119.2 (3)
C1—N1—S3	122.3 (2)	C32—C31—H31	120.4
C3—N2—S6	121.9 (2)	C30—C31—H31	120.4
N1—C1—S2	117.5 (2)	C33—C32—C31	121.2 (3)
N1—C1—S1	126.6 (3)	C33—C32—H32	119.4
S2—C1—S1	115.92 (19)	C31—C32—H32	119.4
F2—C2—F1	108.0 (4)	C32—C33—C34	120.1 (3)
F2—C2—F3	108.0 (4)	C32—C33—H33	120
F1—C2—F3	108.0 (4)	C34—C33—H33	120
F2—C2—S3	112.5 (3)	C33—C34—C29	119.6 (3)
F1—C2—S3	111.5 (3)	C33—C34—H34	120.2
F3—C2—S3	108.7 (3)	C29—C34—H34	120.2
N2—C3—S5	125.6 (2)	C36—C35—C40	119.3 (3)
N2—C3—S4	118.1 (2)	C36—C35—P2	119.8 (3)
S5—C3—S4	116.24 (18)	C40—C35—P2	120.8 (2)
F4—C4—F6	107.6 (4)	C35—C36—C37	119.4 (4)
F4—C4—F5	107.6 (4)	C35—C36—H36	120.3
F6—C4—F5	107.6 (4)	C37—C36—H36	120.3
F4—C4—S6	112.5 (3)	C38—C37—C36	120.1 (4)
F6—C4—S6	111.9 (3)	C38—C37—H37	120
F5—C4—S6	109.4 (3)	C36—C37—H37	120
C6—C5—C10	119.5 (4)	C39—C38—C37	120.9 (4)
C6—C5—P1	120.1 (3)	C39—C38—H38	119.5
C10—C5—P1	120.4 (3)	C37—C38—H38	119.5
C7—C6—C5	119.8 (5)	C38—C39—C40	120.5 (4)
C7—C6—H6	120.1	C38—C39—H39	119.8
C5—C6—H6	120.1	C40—C39—H39	119.8
C6—C7—C8	119.7 (5)	C39—C40—C35	119.8 (4)
C6—C7—H7	120.2	C39—C40—H40	120.1
C8—C7—H7	120.2	C35—C40—H40	120.1
C9—C8—C7	120.5 (5)	C42—C41—C46	119.4 (3)
C9—C8—H8	119.8	C42—C41—P2	122.2 (2)
C7—C8—H8	119.8	C46—C41—P2	118.5 (2)
C8—C9—C10	120.3 (5)	C43—C42—C41	119.8 (3)
C8—C9—H9	119.9	C43—C42—H42	120.1
C10—C9—H9	119.9	C41—C42—H42	120.1
C9—C10—C5	120.3 (4)	C44—C43—C42	120.2 (4)
C9—C10—H10	119.9	C44—C43—H43	119.9
C5—C10—H10	119.9	C42—C43—H43	119.9
C12—C11—C16	119.2 (3)	C43—C44—C45	120.3 (4)
C12—C11—P1	121.7 (3)	C43—C44—H44	119.8
C16—C11—P1	119.0 (3)	C45—C44—H44	119.8
C13—C12—C11	119.6 (4)	C44—C45—C46	120.5 (4)
C13—C12—H12	120.2	C44—C45—H45	119.8
C11—C12—H12	120.2	C46—C45—H45	119.8
C14—C13—C12	121.2 (4)	C45—C46—C41	119.7 (3)
C14—C13—H13	119.4	C45—C46—H46	120.2
C12—C13—H13	119.4	C41—C46—H46	120.2
C13—C14—C15	119.7 (4)	C48—C47—C52	119.4 (3)
C13—C14—H14	120.2	C48—C47—P2	123.8 (2)
C15—C14—H14	120.2	C52—C47—P2	116.7 (2)
C14—C15—C16	120.1 (4)	C49—C48—C47	119.7 (3)
C14—C15—H15	119.9	C49—C48—H48	120.2
C16—C15—H15	119.9	C47—C48—H48	120.2
C15—C16—C11	120.2 (4)	C50—C49—C48	120.7 (3)
C15—C16—H16	119.9	C50—C49—H49	119.7
C11—C16—H16	119.9	C48—C49—H49	119.7
C22—C17—C18	119.6 (3)	C49—C50—C51	120.1 (3)
C22—C17—P1	121.8 (3)	C49—C50—H50	120
C18—C17—P1	118.5 (3)	C51—C50—H50	120
C19—C18—C17	119.1 (4)	C52—C51—C50	119.8 (3)
C19—C18—H18	120.5	C52—C51—H51	120.1
C17—C18—H18	120.5	C50—C51—H51	120.1
C20—C19—C18	120.7 (4)	C51—C52—C47	120.3 (3)
C20—C19—H19	119.6	C51—C52—H52	119.9
C18—C19—H19	119.6	C47—C52—H52	119.9
			
S2—Zn—S1—C1	1.16 (11)	C23—P1—C17—C18	−165.3 (3)
S4—Zn—S1—C1	−130.18 (11)	C11—P1—C17—C18	−44.5 (3)
S5—Zn—S1—C1	124.91 (11)	C22—C17—C18—C19	0.3 (6)
S1—Zn—S2—C1	−1.17 (11)	P1—C17—C18—C19	−178.6 (3)
S4—Zn—S2—C1	134.03 (11)	C17—C18—C19—C20	−0.5 (6)
S5—Zn—S2—C1	−123.66 (11)	C18—C19—C20—C21	0.3 (7)
S2—Zn—S4—C3	123.96 (10)	C19—C20—C21—C22	0.2 (8)
S1—Zn—S4—C3	−125.12 (10)	C18—C17—C22—C21	0.2 (6)
S5—Zn—S4—C3	−0.72 (10)	P1—C17—C22—C21	179.1 (4)
S2—Zn—S5—C3	−127.47 (10)	C20—C21—C22—C17	−0.4 (8)
S1—Zn—S5—C3	133.86 (10)	C17—P1—C23—C28	73.9 (3)
S4—Zn—S5—C3	0.72 (10)	C5—P1—C23—C28	−166.0 (3)
O1—S3—N1—C1	−65.2 (3)	C11—P1—C23—C28	−43.8 (3)
O2—S3—N1—C1	74.6 (3)	C17—P1—C23—C24	−103.9 (3)
C2—S3—N1—C1	−175.9 (3)	C5—P1—C23—C24	16.1 (3)
O3—S6—N2—C3	70.0 (3)	C11—P1—C23—C24	138.3 (3)
O4—S6—N2—C3	−70.8 (3)	C28—C23—C24—C25	1.0 (6)
C4—S6—N2—C3	−180.0 (3)	P1—C23—C24—C25	178.9 (3)
S3—N1—C1—S2	−175.78 (19)	C23—C24—C25—C26	−0.9 (7)
S3—N1—C1—S1	4.8 (5)	C24—C25—C26—C27	−0.1 (6)
Zn—S2—C1—N1	−177.8 (3)	C25—C26—C27—C28	0.9 (6)
Zn—S2—C1—S1	1.71 (16)	C24—C23—C28—C27	−0.2 (5)
Zn—S1—C1—N1	177.7 (3)	P1—C23—C28—C27	−178.1 (3)
Zn—S1—C1—S2	−1.71 (16)	C26—C27—C28—C23	−0.7 (6)
O1—S3—C2—F2	−176.8 (3)	C41—P2—C29—C30	129.3 (2)
O2—S3—C2—F2	59.5 (3)	C35—P2—C29—C30	9.2 (3)
N1—S3—C2—F2	−57.3 (3)	C47—P2—C29—C30	−112.9 (2)
O1—S3—C2—F1	−55.3 (3)	C41—P2—C29—C34	−54.8 (3)
O2—S3—C2—F1	−179.0 (3)	C35—P2—C29—C34	−175.0 (2)
N1—S3—C2—F1	64.2 (3)	C47—P2—C29—C34	63.0 (3)
O1—S3—C2—F3	63.6 (4)	C34—C29—C30—C31	−2.3 (4)
O2—S3—C2—F3	−60.1 (4)	P2—C29—C30—C31	173.6 (2)
N1—S3—C2—F3	−176.9 (3)	C29—C30—C31—C32	1.1 (5)
S6—N2—C3—S5	−0.1 (4)	C30—C31—C32—C33	0.6 (5)
S6—N2—C3—S4	179.99 (16)	C31—C32—C33—C34	−1.2 (5)
Zn—S5—C3—N2	179.0 (3)	C32—C33—C34—C29	0.0 (5)
Zn—S5—C3—S4	−1.06 (15)	C30—C29—C34—C33	1.7 (5)
Zn—S4—C3—N2	−179.0 (2)	P2—C29—C34—C33	−174.2 (2)
Zn—S4—C3—S5	1.07 (15)	C41—P2—C35—C36	−10.6 (3)
O3—S6—C4—F4	178.5 (3)	C47—P2—C35—C36	−129.2 (2)
O4—S6—C4—F4	−57.0 (3)	C29—P2—C35—C36	110.5 (3)
N2—S6—C4—F4	61.0 (3)	C41—P2—C35—C40	172.0 (2)
O3—S6—C4—F6	57.3 (4)	C47—P2—C35—C40	53.3 (3)
O4—S6—C4—F6	−178.3 (3)	C29—P2—C35—C40	−67.0 (3)
N2—S6—C4—F6	−60.3 (4)	C40—C35—C36—C37	−0.5 (5)
O3—S6—C4—F5	−62.0 (4)	P2—C35—C36—C37	−178.0 (3)
O4—S6—C4—F5	62.5 (4)	C35—C36—C37—C38	−1.1 (5)
N2—S6—C4—F5	−179.5 (3)	C36—C37—C38—C39	1.9 (6)
C17—P1—C5—C6	174.8 (4)	C37—C38—C39—C40	−1.1 (6)
C23—P1—C5—C6	55.3 (4)	C38—C39—C40—C35	−0.6 (5)
C11—P1—C5—C6	−67.4 (4)	C36—C35—C40—C39	1.3 (5)
C17—P1—C5—C10	−2.4 (4)	P2—C35—C40—C39	178.8 (2)
C23—P1—C5—C10	−122.0 (3)	C35—P2—C41—C42	102.1 (3)
C11—P1—C5—C10	115.4 (3)	C47—P2—C41—C42	−136.1 (3)
C10—C5—C6—C7	0.8 (8)	C29—P2—C41—C42	−16.8 (3)
P1—C5—C6—C7	−176.5 (5)	C35—P2—C41—C46	−78.2 (3)
C5—C6—C7—C8	0.9 (10)	C47—P2—C41—C46	43.6 (3)
C6—C7—C8—C9	−2.9 (11)	C29—P2—C41—C46	162.9 (3)
C7—C8—C9—C10	3.1 (10)	C46—C41—C42—C43	0.2 (5)
C8—C9—C10—C5	−1.3 (8)	P2—C41—C42—C43	179.9 (3)
C6—C5—C10—C9	−0.7 (7)	C41—C42—C43—C44	1.1 (6)
P1—C5—C10—C9	176.6 (4)	C42—C43—C44—C45	−0.7 (7)
C17—P1—C11—C12	131.2 (3)	C43—C44—C45—C46	−1.1 (7)
C5—P1—C11—C12	11.2 (3)	C44—C45—C46—C41	2.5 (6)
C23—P1—C11—C12	−109.4 (3)	C42—C41—C46—C45	−2.0 (5)
C17—P1—C11—C16	−45.4 (3)	P2—C41—C46—C45	178.3 (3)
C5—P1—C11—C16	−165.4 (3)	C41—P2—C47—C48	−124.1 (3)
C23—P1—C11—C16	74.1 (3)	C35—P2—C47—C48	−3.9 (3)
C16—C11—C12—C13	0.3 (5)	C29—P2—C47—C48	115.4 (3)
P1—C11—C12—C13	−176.2 (3)	C41—P2—C47—C52	55.2 (3)
C11—C12—C13—C14	−0.3 (6)	C35—P2—C47—C52	175.4 (3)
C12—C13—C14—C15	−0.4 (6)	C29—P2—C47—C52	−65.3 (3)
C13—C14—C15—C16	1.2 (7)	C52—C47—C48—C49	0.7 (5)
C14—C15—C16—C11	−1.2 (7)	P2—C47—C48—C49	179.9 (3)
C12—C11—C16—C15	0.4 (5)	C47—C48—C49—C50	0.6 (5)
P1—C11—C16—C15	177.1 (3)	C48—C49—C50—C51	−0.9 (6)
C5—P1—C17—C22	−103.0 (3)	C49—C50—C51—C52	−0.1 (6)
C23—P1—C17—C22	15.8 (4)	C50—C51—C52—C47	1.4 (6)
C11—P1—C17—C22	136.6 (3)	C48—C47—C52—C51	−1.7 (5)
C5—P1—C17—C18	75.9 (3)	P2—C47—C52—C51	179.0 (3)

### Hydrogen-bond geometry (Å, °)

**Table:**

*D*—H···*A*	*D*—H	H···*A*	*D*···*A*	*D*—H···*A*
C9—H9···O1	0.93	2.68	3.424 (4)	138.
C31—H31···O4	0.93	2.62	3.470 (4)	153.
C33—H33···O2^i^	0.93	2.47	3.283 (4)	145.

Symmetry codes:  (i) *x*, −*y*+1/2, *z*−1/2.
